# *Helicobacter pylori* Outer Membrane Vesicles: Biogenesis, Composition, and Biological Functions

**DOI:** 10.7150/ijbs.94156

**Published:** 2024-07-15

**Authors:** Jiao Li, Tingting Liao, Eng Guan Chua, Mingming Zhang, Yalin Shen, Xiaona Song, Barry J. Marshall, Mohammed Benghezal, Hong Tang, Hong Li

**Affiliations:** 1 Center of Infectious Diseases, West China Hospital, Sichuan University, Chengdu, China.; 2 Laboratory of Infectious and Liver Diseases, Institute of Infectious Diseases, West China Hospital of Sichuan University, Chengdu 610041, China.; 3 QuTEM AB, Gävlegatan 22, 11330 Stockholm, Sweden.; 4 Helicobacter Research Laboratory, The Marshall Centre for Infectious Disease Research and Training, University of Western Australia, Nedlands WA 6009, Australia.

**Keywords:** *Helicobacter pylori*, outer membrane vesicles, biogenesis, composition, biological functions

## Abstract

*Helicobacter pylori* has been recognized not only as a causative agent of a spectrum of gastroduodenal diseases including chronic gastritis, peptic ulcer, mucosa-associated lymphoid tissue lymphoma, and gastric cancer, but also as the culprit in several extra-gastric diseases. However, the association of *H. pylori* infection with extra-gastric diseases remains elusive, prompting a reevaluation of the role of *H. pylori*-derived outer membrane vesicles (OMVs). Like other gram-negative bacteria, *H. pylori* constitutively sheds biologically active OMVs for long-distance delivery of bacterial virulence factors in a concentrated and protected form, averting the need of direct bacterial contact with distant host cells to induce extra-gastric diseases associated with this gastric pathogen. Additionally, *H. pylori*-derived OMVs contribute to bacterial survival and chronic gastric pathogenesis. Moreover, the immunogenic activity, non-replicable nature, and anti-bacterial adhesion effect of* H. pylori* OMVs make them a desirable vaccine candidate against infection. The immunogenic potency and safety concerns of the OMV contents are challenges in the development of *H. pylori* OMV-based vaccines. In this review, we discuss recent advances regarding *H. pylori* OMVs, focusing on new insights into their biogenesis mechanisms and biological functions.

## Introduction

*Helicobacter pylori*, a gram-negative and spiral-shaped bacterium, colonizes the human gastric mucosa of about half of the world's population 1. *Helicobacter pylori* is usually acquired in childhood and can persist lifelong within the human stomach unless treated with antibiotics. Upon infection with *H. pylori*, the host responds by initiating inflammation of the gastric mucosa, causing gastritis, but failing to eradicate the pathogen. *H. pylori*-induced gastritis is usually asymptomatic, but prolonged infection is a major risk factor for the development of peptic ulcer, mucosa-associated lymphoid tissue (MALT) lymphoma, and gastric cancer 2, 3. Therefore, *H. pylori* is listed as a group Ⅰ carcinogen by the World Health Organization (WHO) 4.

In addition to gastric diseases, emerging evidence indicates that *H. pylori* infection is associated with various extra-gastric diseases, such as cardiovascular diseases 5, diabetes 6, liver diseases 7, and Alzheimer's disease 8. However, the mechanisms underlying the remote effects of *H. pylori* infection remain largely unknown. As an extracellular bacterium with poor invasiveness, most *H. pylori* reside in the gastric mucus layer, with only a small proportion attached to the surface of epithelial cells 9, 10. Since there is no evidence that *H. pylori* can enter the blood circulation 11, *H. pylori*-derived virulence factors, especially the outer membrane vesicles (OMVs), may play an important role in the development of the reported extra-gastric diseases. Shedding spherical and membranous OMVs, ranging between 20-300 nm in diameter, is a common feature of virtually all gram-negative bacteria 12. Emerging evidence indicates that OMVs from the host microbiota, particularly the commensal bacteria in the gastrointestinal tract, can enter blood circulation 13. Compared with soluble secretion pathways, OMVs provide a relatively superior secretion option for pathogens to deliver virulence factors in a highly concentrated form, whilst offering additional protection from host proteases during travelling to distant organs and cells 14. *Helicobacter pylori* constitutively sheds OMVs both *in vivo* and *in vitro* 15-17. A recent study showed that the transcriptomic changes induced by *H. pylori* OMVs in MKN74 gastric adenocarcinoma cell line were largely similar to those triggered by the parental bacteria 18, indicating that OMVs may largely contribute to or amplify the pathogenic effects induced by the bacterium itself. Moreover, the detection of *H. pylori*-derived OMVs in the sera of mice infected with *H. pylori* provided further evidence for the potential involvement of *H. pylori* OMVs in extra-gastric diseases 19.

Apart from the role as a delivery vehicle for virulence factors, OMVs exhibit a large variety of other biological roles, such as horizontal gene transfer, nutrient acquisition, bacterial biofilm formation, and host immune response modulation 20, 21. OMVs contain abundant bacterial antigens, including lipids, proteins, carbohydrates, and nucleic acids. By eliciting and modulating immune responses, as well as preventing pathogen localization and proliferation, OMVs may play an anti-infection role in attenuating and even preventing pathogen-associated diseases 22. This is supported by the good safety and efficacy profiles of OMV-based vaccines against *Neisseria meningitidis* 22-24. However, the potential use of *H. pylori* OMVs as vaccines against *H. pylori* infection is just at the early stage.

In this review, we discuss recent advances regarding *H. pylori* OMVs biogenesis and composition with a special focus on their pathological roles in gastric and extra-gastric diseases, and their potential use as vaccines.

## OMV biogenesis

### Models for OMV biogenesis in other gram-negative bacteria

**Figure 1:**
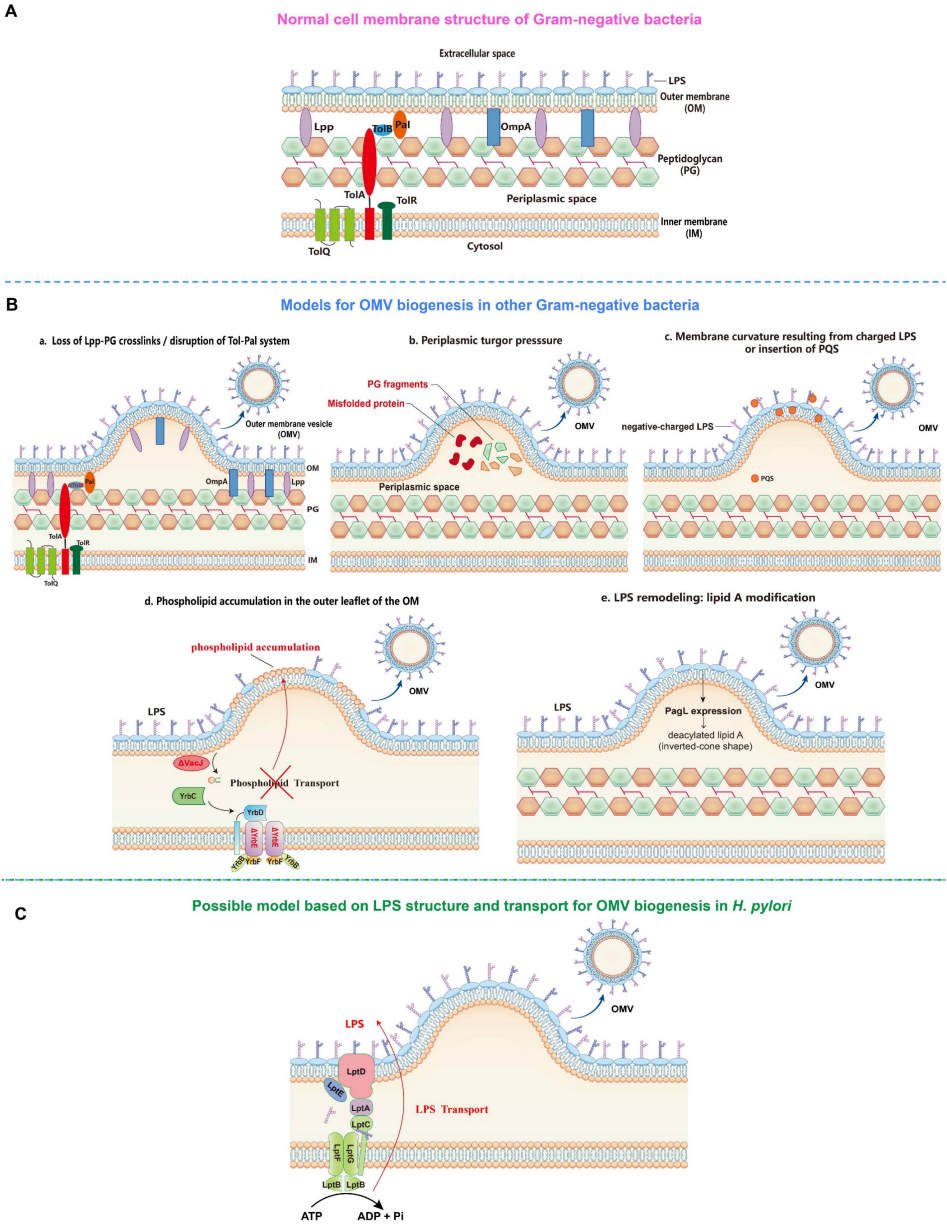
** OMV biogenesis models** (By Figdraw).** A**. Cell membrane structure of gram-negative bacteria consists of inner membrane (IM), periplasmic space containing peptidoglycan (PG) and outer membrane (OM).** B**. OMV biogenesis models in other gram-negative bacteria: **a.** Loss of covalent crosslinks between the OM and PG or disruption of non-covalent crosslinks through Tol-Pal system; **b.** Accumulation of misfolded proteins and PG fragments in the periplasmic space causes turgor pressure; **c.** Electrostatic repulsion between negative-charged lipopolysaccharide (LPS) molecules or insertion of *Pseudomonas* quinolone signal (PQS) into the OM induce membrane curvature; **d**. Phospholipid accumulation in the outer leaflet of the OM resulted from the disruption within VacJ/Yrb ABC transport system; **e.** LPS remodeling mediated by PagL expression-induced deacylation of lipid A. **C**. Hypothetical OMV biogenesis model based on LPS structure and transport in *Helicobacter pylori*.

The first model proposes that the formation of OMVs is due to the loss of covalent or non-covalent crosslinks between the outer membrane (OM) and the underlying peptidoglycan layer (**Figure 1**). For instance, the lack or reduction of covalent crosslinks of lipoprotein (Lpp) or outer membrane protein A (OmpA) with the peptidoglycan layer allows a faster growth rate of the OM relative to the peptidoglycan layer, which then leads to the detachment and release of protruded OM as OMVs in several gram-negative bacteria including *Escherichia coli*, *Acinetobacter baumannii*, and *Salmonella* spp. 25-28. In addition, the disruption of one or more components of the Tol-Pal system (a protein complex comprising the inner membrane proteins TolQ-TolR-TolA, the periplasmic protein TolB, and the outer membrane protein Pal), which is involved in non-covalent interaction with the peptidoglycan, has also been shown to significantly increase OMV production in *E. coli*, *Salmonella choleraesuis*, *Shigella flexneri*, and *Bordetella pertussis* 29-33.

The second model proposes that turgor pressure, caused by the accumulation of peptidoglycan fragments or misfolded proteins in the periplasmic space, induces the bulging and pinching-off of the bacterial OM (**Figure 1**). This model is supported by the detection of low molecular weight muramic acid, a precursor of peptidoglycan, in OMVs isolated from *Porphyromonas gingivalis* 34, and the deletion of peptidoglycan amidase in the same bacterial species that has resulted in the increase of OMV production, possibly due to the disruption of the peptidoglycan recycling process and thus the buildup of turgor pressure following the periplasmic accumulation of peptidoglycan fragments 35. Furthermore, McBroom *et al.* demonstrated that the extent of vesiculation is correlated with the level of protein accumulation in the cell envelope 36. Another study also reported that the inactivation of DegQ periplasmic serine protease has led to abnormal accumulation of proteins in the periplasmic space, causing increased OMV production in *Shewanella oneidensi* 37.

The third model involves the induction of membrane curvature, which is contributed mainly by lipopolysaccharide (LPS) and *Pseudomonas* quinolone signal (PQS) (**Figure 1**). Bacterial LPS, typically composed of three domains including lipid A, core oligosaccharide, and the outermost O-antigen polysaccharide, is a major constituent of the outer leaflet of the OM of gram-negative bacteria 38. The LPS of most gram-negative bacteria is negatively charged due to the presence of anionic phosphate groups in the lipid A and inner core. The negatively charged LPS molecules are crosslinked by divalent cations, such as Ca^2+^ and Mg^2+^, to stabilize the OM 39. Thus, the imbalance of the ionic interaction may lead to the strong electrostatic repulsion between neighbouring LPS molecules, inducing membrane curvature and subsequent OMV formation. This is supported by the finding that the treatment of cells with divalent cation chelator EDTA enhanced the release of OMVs, whereas the addition of exogenous Mg^2+^ suppressed OMV formation 40. It has also been found that OMVs from *Pseudomonas aeruginosa* consist primarily of the negatively charged B-band LPS, but not the neutral A-band LPS present in the OM 41, 42. Similarly, in *P. gingivalis*, only the negatively charged LPS has been found in OMVs 43, suggesting the involvement of charge-to-charge repulsion by LPS in OMV formation. Interestingly, PQS, a membrane curvature-inducing and LPS-binding signaling molecule, is not only packaged into OMVs, but also required for OMV production in *P. aeruginosa* 44, 45. It has been shown that PQS stimulates OMV biogenesis through its interaction with LPS. By sequestering divalent cations, PQS enhances anionic repulsions between LPS molecules, causing a bilayer couple effect that induces membrane curvature and the budding of OMVs 46, 47. Of note, the PQS-based model is species-specific as PQS is only produced by *P. aeruginosa* 22.

The fourth model involves the VacJ/Yrb ABC (ATP-binding cassette) transport system, a phospholipid (PL) transporter, which functions to prevent PL accumulation in the outer leaflet of the OM by transporting PLs retrogradely from the OM to the inner membrane (IM), thereby maintaining the lipid asymmetry in the OM 48 (**Figure 1**). It has been reported that the deletion or downregulated expression of VacJ or YrbE resulted in increased OMV production in several distantly related bacteria, including *Haemophilus influenzae*, *Vibrio cholerae*, and* E. coli* 48. Lipidomic analyses indicated that phosphatidylethanolamine (PE) was the most dominant PL species in OM and OMVs preparations, and the total PE content of OMVs derived from the transporter mutants was two-fold higher than that of the wild-type OMVs. The accumulation of PLs in the outer leaflet of the OM induced by deletion or reduced expression of the VacJ/Yrb ABC transport system induces an outward bulging of the OM, which pinches off to form OMVs 48.

The fifth model involves LPS lipid A deacylation-mediated OM remodeling (**Figure 1**). It has been reported that the expression of the “latent” lipid A deacylase PagL in intracellular *Salmonella typhimurium* results in hypervesiculation without inducing an envelope stress response 49. Mass spectrometry analysis further revealed considerable differences in the patterns of lipid A between OM and OMVs, with deacylated lipid A accumulating exclusively in OMVs. In contrast, the *S. typhimurium* Δ*pagL* strain displayed a significant reduction in intracellular OMV production compared to the wild-type strain. It was proposed that intracellular *S. typhimurium* PagL is activated to deacylate lipid A, leading to a decrease in hydrophobic cross-section area. The deacylated lipid A adopts an inverted-cone shaped structure, leading to membrane curvature and OMV formation 49.

### Possible OMV biogenesis model in *H. pylori*

Although the mechanistic models for OMV biogenesis are increasingly being unraveled, whether these models are applicable to the OMV biogenesis in *H. pylori* remains to be further investigated. Interestingly, a study demonstrated that knocking out *hp0044* and *hp1275* genes responsible for GDP-fucose biosynthesis have led to a truncated LPS (truncating from the conserved O-antigen Trio fucose residue) and altered protein sorting into OMVs 50. In addition, a recent study showed that inactivation of HP0860 responsible for ADP-heptose biosynthesis also caused truncation of the LPS core and altered protein sorting into OMVs 51. These results suggest that *H. pylori* LPS may play an important role in OMV cargo sorting and biogenesis. Considering that OMV production requires a significant energy cost, whereas ATP or other energy sources directly at the site of budding are not available 52, we hypothesize that the ATP used to export a continuous stream of newly synthesized LPS from the IM to the outer leaflet of the OM through the transenvelope Lpt machinery comprising seven LPS transport proteins (LptA-G) is likely to be source of energy for OMV biogenesis in *H. pylori* 53 **(Figure 1)**.

## Composition of *H. pylori* OMVs

### Lipids and LPS

The total lipid content of *H. pylori* consists of about 70% phospholipids, up to 25% of unique cholesteryl glucosides (CGs), and the rest being neutral lipids 54. In another study, it was shown that the lipid profile of *H. pylori* OMVs is highly similar to that of the OM, with PE being the most abundant phospholipid detected in *H. pylori* OMVs and then followed by cardiolipin. Additional phospholipids found in *H. pylori* OMVs include lyso-PE, phosphatidylglycerol (PG), lyso-PG, phosphatidylcholine (PC) and lyso-PC 55.

Most gram-negative bacterial OMVs characterized to date are enriched with LPS that is structurally identical to the parental bacteria 9, 56, 57. The *H. pylori* LPS contains lipid A, core oligosaccharide, and the O-antigen. The O-antigen portion contains Lewis antigen composed of a Gal-GlcNAc backbone chain, which is divided into two types based on the linkage. Namely, type 1 composed of Gal-β-(1,3)-GlcNAc forms Lewis a (Le^a^) and Lewis b (Le^b^), and type 2 composed of Gal-β-(1,4)-GlcNAc forms Lewis x (Le^x^) and Lewis y (Le^y^) 58. It was reported that the abundance of LPS in *H. pylori* OMVs is influenced by the iron availability in the parental bacteria growth conditions, since less and shorter LPS was detected in OMVs from iron-limiting bacteria than that from iron-replete bacteria. More specifically, structural analysis and serological detection revealed that iron-limiting bacterial whole cells and OMVs exhibited reduced Le^y^ expression 59.

### Proteins

A mass spectrometry (MS)-based proteomic analysis of OMVs isolated from *H. pylori* strain J99 identified 162 distinct proteins, of which 30.86% (50), 21.6% (35), 7.4% (12) and 4.9% (8) were known or predicted OM, cytoplasmic, periplasmic and IM proteins, respectively, and proteins with no confirmed or predicted subcellular localization accounting for 29.6% 60. In another study, more than 300 different *H. pylori* proteins were identified in the OMV proteome of strain CCUG17875. Interestingly, OM proteins constituted only 16% of the OMV proteome 55. More recently, Wei *et al.* identified 436 proteins in the reference strain NCTC11637 OMVs and 372 proteins in the clinical strain Hp-400 OMVs 61. The discrepancies exhibited by these studies lead to the conclusion that *H. pylori* OMVs show great heterogeneity in their protein composition, which may be determined by strain genotypes, different bacterial growth conditions, growth stages, OMV preparation, and the sensitivity of MS methods used for proteomic analysis 62. Of note, a recent study revealed that the OMV size can determine its protein cargo content, as smaller OMVs contained significantly fewer and less diverse proteins compared to larger ones, and most *H. pylori* adhesins were packaged within larger OMVs 63.

Among the protein components of *H. pylori*, CagA and VacA cytotoxins are the most studied virulence factors 64. VacA is a pore-forming toxin that induces vacuolation in gastric epithelial cells 65, 66. Although it is an autotransporter protein, VacA has also been identified in *H. pylori* OMVs. By electron microscopy and immunocytochemistry, VacA was confirmed to be released by *H. pylori* in both free-soluble and OMV-associated forms, both of which were observed to be internalized by MKN28 gastric adenocarcinoma cell line* in vitro*, and were detected in the gastric mucosa isolated from *H. pylori*-infected patients 67. Quantitative analysis revealed that OMV-associated VacA accounted for about 25% of total VacA, with the remaining 75% being free-soluble 68. CagA cytotoxin, also known for its role as an oncoprotein, can induce a “hummingbird” phenotype in gastric epithelial cells 69. Typically, CagA is injected into host cells by the bacteria through the type IV secretion system (T4SS) 70. Intriguingly, CagA was found to be associated with the surface of *H. pylori* OMVs, suggesting that OMVs might provide an alternative mechanism for *H. pylori* to transport CagA directly into host cells independently of the T4SS 55, 71. Furthermore, additional proteins involved in *H. pylori* colonization and virulence, such as SabA, BabA, AlpA, AlpB, OipA, and HopZ adhesins, and several other enzymes including urease, catalase and HtrA serine protease were identified in the OMVs 55, 60, 61, 71-73.

### Peptidoglycan and nucleic acids

Peptidoglycan component can be found in *H. pylori* OMV as demonstrated by Kaparakis *et al.* that OMVs isolated from *H. pylori* strain 251 were shown to contain approximately 0.3-0.5 ng of the muramic acid moiety of peptidoglycan, per μg of OMV protein 74. Besides, genetic materials including extracellular DNA (eDNA) and small non-coding RNA (sncRNA) have also been detected in OMVs from multiple gram-negative bacteria 56, 75. In *H. pylori*, eDNA implicated in communication between bacteria was detected on the surface of OMVs from *H. pylori* strain NCTC 11639 76. Moreover, Zhang* et al.* identified the presence of regulatory sncRNAs (sR-2509025 and sR-989262) in OMVs isolated from *H. pylori* strain J99 by RNA sequencing 77.

## Biological functions of *H. pylori* OMVs

### *H. pylori* OMVs favor bacterial biofilm formation and resistance to antimicrobials

Biofilm formation represents a survival strategy by which bacteria are protected from the attack of both host immune system and antimicrobials, thus contributing to bacterial persistence 78, 79. Notably, the ability of* H. pylori* to form biofilms on abiotic surfaces *in vitro* as well as on human gastric mucosa* in vivo* has been well characterized 78, 80. The favorable role of OMVs in *H. pylori* biofilm formation was confirmed in *in vitro* studies. Yonezawa *et al.* demonstrated that biofilm formation of *H. pylori* was dependent on the cell-cell aggregation mediated by OMVs 81. In addition, the presence of OMVs was detected in the biofilm extracellular polymeric substance matrix of TK1402 clinical isolate with strong biofilm-forming ability, and the addition of TK1402-derived OMVs to *H. pylori* cultures resulted in remarkably enhanced biofilm formation 81. By further analyzing the protein profiles of OMVs from different *H. pylori* strains with strong or weak biofilm-forming ability, an uncharacterized 22-KDa protein and the outer membrane protein AlpB were identified as crucial factors contributing significantly to the biofilm formation in the strain TK1402 82, 83. In addition to protein molecules, eDNA, mainly associated with the surface of OMVs, appears to play a pivotal role during the development of *H. pylori* biofilms 76. *Helicobacter pylori* produces OMVs in both biofilm (bOMVs) and planktonic (pOMVs) phenotypes. In *H. pylori* strain NCTC11639, bOMVs were found to have a broader size range, more negative charges, increased OMV aggregation, as well as a four-fold increase in eDNA as compared with pOMVs, suggesting that *H. pylori* bOMVs could prevent the degradation of eDNA. The eDNA may then act as a bridge to facilitate OMV-OMV and cell-cell aggregation, ultimately promoting biofilm formation 76. More recently, the α-class carbonic anhaydrase (α-CA), a periplasmic enzyme essential for the acid acclimation of *H. pylori* within human stomach, was also detected in bOMVs and pOMVs of four *H. pylori* strains, with a higher level seen in pOMVs than in bOMVs. Moreover, α-CA was confirmed to induce the release of eDNA, thereby stabilizing the biofilm formation 84. Correspondingly, carvacrol and thymol, inhibitors of α-CA, could prevent *H. pylori* OMV production and biofilm development via the inhibition of eDNA release 85. Taken together, *H. pylori*-derived OMVs are both “components” and “boosters” of bacterial biofilm, which represents a survival strategy exploited by the microorganism to protect itself against the attack of both host immune system and antimicrobials 86.

Apart from facilitating biofilm formation, OMVs confer bacterial resistance to antimicrobials in two possible ways: 1) OMV-mediated transfer of antibiotic resistance genes between bacteria; 2) OMVs acting as decoys to bind to or absorb antimicrobials 14, 56. For instance, in *E. coli*, the use of a hypervesiculating mutant or the addition of OMVs enhanced immediate resistance to the antimicrobial peptides polymyxin B and colistin 87. Similarly, OMVs from *Pseudomonas syringae* reduced the levels of colistin and melittin in solution by sequestering these compounds 88. In *H. pylori*, it was found that the addition of purified OMVs from strain 60190 facilitated *H. pylori* survival upon exposure to antimicrobial peptide LL-37 as well as clarithromycin 89. Given that clarithromycin is a hydrophobic antibiotic that enters bacterial cells via lipid-mediated passive diffusion 31, 32, and it is also a macrolide antibiotic that can bind directly to lipid membranes 33, *H. pylori* OMVs of lipid bilayer may act as a decoy sequestering clarithromycin that would otherwise diffuse into bacterial cells 89.

**Figure 2:**
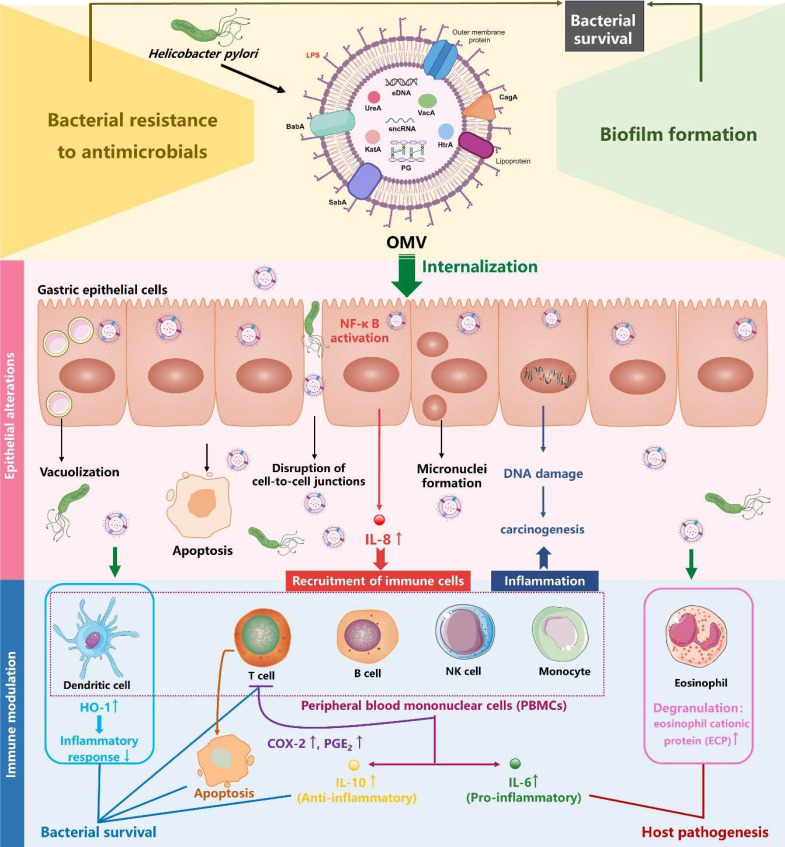
**Role of *H. pylori* OMVs in bacterial survival and host gastric pathogenesis** (created with BioRender.com). On the one hand, *H. pylori* OMVs promote biofilm formation and enhance bacterial resistance to antimicrobials, both of which synergistically admit *H. pylori* to survive and aggravate the chronic infection. On the other hand, *H. pylori* OMVs containing multiple virulence factors can be internalized by gastric epithelial cells and induced a plethora of pathological effects: elevation of proinflammatory factor IL-8, apoptosis, disruption of gastric epithelial cell-to-cell junctions, vacuolization, micronuclei formation. These alterations in gastric epithelial cells contribute to the development of gastric diseases. Additionally, *H. pylori* OMVs can modulate immune cells within the lamina propria, either suppressing the immune response to promote bacterial survival or stimulating the inflammatory response, thus contributing to host pathogenesis.

Therefore, the OMVs produced by *H. pylori* play a crucial role in its survival by promoting *H. pylori* biofilm formation and/or acting as a decoy to sequester host-derived antimicrobials or antibiotics (**Figure 2**).

### *H. pylori* OMVs contribute to the pathogenesis of gastric and extra-gastric diseases

#### Uptake of *H. pylori* OMVs by host cells

Uptake of *H. pylori* OMVs by host cells is the initial step required for delivering bacterial virulence factors into host cells and exerting pathological effects. By utilizing an *in vivo* imaging system, it was found that orally administrated *H. pylori* OMVs remained in the stomach of mice for at least 24 h and could enter gastric epithelial cells 17. *In vitro* assays also observed that *H. pylori* OMVs adhered to and were internalized by primary human antral epithelial cells, as well as by AGS gastric adenocarcinoma cell line 90, 91. Fluorescent confocal microscopy revealed that internalized vesicles colocalized with markers for early endosomes and lysosomes, suggesting their entry through the endocytic pathway 91, 92. Additionally, UreA was observed to localize to the cytoplasm and nucleus of AGS cells incubated with OMVs from *H. pylori* strain 26695, reinforcing the entry of* H. pylori* OMVs into host cells and subsequent release of virulence factors 93. Cellular uptake of *H. pylori* OMVs has been reported to occur through both clathrin-dependent and clathrin-independent pathways 92, with clathrin-mediated endocytosis identified as the primary mechanism 94. For instance, treatment of AGS cells with chlorpromazine, a known inhibitor of clathrin-mediated endocytosis, reduced the uptake of* H. pylori* OMVs 92, 95. Disruption of lipid rafts in AGS cells using Fumonisin B1, an inhibitor of sphingomyelin, or methyl-β-cyclodextrin (MβCD), a cholesterol-depleting agent, also reduced OMV uptake, suggesting the involvement of lipid raft-mediated endocytosis 55, 74. Additionally, caveolin- and dynamin-mediated endocytosis, macropinocytosis, and phagocytosis are also identified as important mechanisms mediating the entry of *H. pylori* OMVs into host cells 63, 91. Notably, the size of *H. pylori* OMVs was reported to determine the mechanisms of their uptake by host cells. Vesicles smaller than 100 nm are typically taken up through endocytosis, whereas the vesicles larger than 200 nm are usually taken up through macropinocytosis and phagocytosis 96. Moreover, Parker *et al.* reported that the presence of VacA in OMVs significantly enhanced vesicle association with host cells. They found that the internalization of VacA^+^ OMVs was less inhibited by chlorpromazine compared to VacA^-^ OMVs, suggesting that VacA allows vesicles to use more than one pathway of internalization 95.

#### Pathogenic effects of *H. pylori* OMVs on gastric epithelial cells

It has been well established that *H. pylori* OMVs stimulate IL-8 production by gastric epithelial cells in a dose-dependent manner, both *in vivo* and *in vitro* 17, 61, 97. IL-8 serves as a pro-inflammatory cytokine and chemokine, triggering the recruitment of immune cells such as macrophages, neutrophils, and T lymphocytes to the infected tissues, thereby inducing an intense mucosal inflammatory response 98. Kaparakis *et al.* demonstrated that the OMV-mediated delivery of peptidoglycan, rather than LPS, induced cytosolic NOD1-dependent NF-κB activation and subsequent IL-8 production in AGS cells. Notably, microinjection of peptidoglycan into AGS cells failed to initiate a NOD1-dependent inflammatory response, highlighting the crucial role of *H. pylori* OMVs in the delivery of bacterial peptidoglycan to induce host cell inflammation 74. Interestingly, it was also demonstrated in another study that OMVs were involved in the delivery of LPS into host cell cytosol, triggering caspase-11-dependent pyroptotic cell death and IL-1 responses 57. Future study is needed to investigate whether* H. pylori* LPS can rely on OMVs to gain access into cytosol for caspase-11 activation.

*H. pylori* OMVs have also been reported to induce apoptosis in AGS cells through the activation of caspase-8 and caspase-3, independently of the mitochondrial pathway, as indicated by the absence of cytochrome c release 99. Moreover, OMVs can enhance the carcinogenic potential of *H. pylori* as increased micronuclei formation (a cellular event associated with carcinogenesis) was observed in AGS cells when treated with OMVs from *H. pylori* strain 60190 100. Additionally, a transcriptomic study demonstrated that OMVs from *H. pylori* strain 26695 induced transcriptomic remodeling of MKN74 cells, characterized by downregulation of cell cycle, DNA replication, and DNA repair 18. This remodeling may lead to accumulated DNA mutations and genome instability, predisposing cells to carcinogenesis. Consistently, a proteomic study reported that GES-1 human normal gastric epithelial cell line treated with OMVs from *H. pylori* strains NCTC11637 and Hp400 exhibited proteomic changes related to cancer signaling pathways, including increased expression of the pro-carcinogenic proteins VTN and complement C3 61. Notably, OMV-induced transcriptomic and proteomic alterations in MKN74 and GES-1 cells largely coincided with *H. pylori*-induced alterations 18, 61, implying that *H. pylori* induces pathogenicity in gastric epithelial cells in part by producing OMVs. Collectively, *H. pylori* OMVs contribute to the development of gastric diseases by promoting inflammation, apoptosis, and tumorigenesis in gastric epithelial cells (**Figure 2**).

#### Immunomodulatory effects of *H. pylori* OMVs on immune cells within gastric lamina propria

The ability of *H. pylori* OMVs to transmigrate across gastric epithelial monolayers allows these vesicles to directly deliver antigens and virulence factors to immune cells infiltrating the lamina propria, which is triggered by *H. pylori* infection 70. For instance, an *in vitro* study showed that eosinophils treated with *H. pylori* OMVs or co-cultured with OMV-exposed gastric epithelial cells were activated and underwent degranulation, releasing cytotoxic granule proteins such as eosinophil cationic protein (ECP) which exacerbate local inflammatory responses and tissue damage 101. Additionally, Winter *et al.* reported that OMVs from *H. pylori* stains SS1 and 60190 strongly triggered the production of both pro-inflammatory cytokine IL-6 and anti-inflammatory cytokine IL-10 by human peripheral blood mononuclear cells (PBMCs) in a dose-dependent manner, suggesting a delicate role for *H. pylori* OMVs in the modulation of host cell innate immune response 102. Another study revealed that exposure of both human PBMC-derived dendritic cells (DCs) and murine bone marrow-derived DCs to *H. pylori* OMVs upregulated heme oxygenase-1 (HO-1) expression via the activation of Akt-Nrf2 and mTOR-NF-κB signaling pathways, potentially inhibiting the maturation of DCs to help regulate inflammatory responses during *H. pylori* infection 103. In terms of T cells, *H. pylori* OMVs induced apoptosis in both Jurkat T cell line and native CD4^+^ T cells, suggesting a role for *H. pylori* OMVs in the repression of T cell immunity 102. Consistently, a more recent study revealed that *H. pylori* OMVs indirectly inhibited T cell responses by inducing cyclooxygenase-2 expression in monocytes, thereby increasing levels of prostaglandin-E2 and IL-10 104. Taken together, these findings suggest that *H. pylori* OMVs are associated with gastric pathologies and the bacterium's persistence in the gastric mucosa through exerting immunomodulatory effects on immune cells within the lamina propria (**Figure 2**).

#### Extra-gastric pathogenic effects of *H. pylori* OMVs

Owing to their nanometric structure with the ability to serve as delivery vehicles for their cargo virulence factors, *H. pylori*-derived OMVs have increasingly been recognized as a mechanistic link between *H. pylori* infection and several extra-gastric diseases, such as liver fibrosis, Alzheimer's disease, and atherosclerosis 105-108.

**Figure 3:**
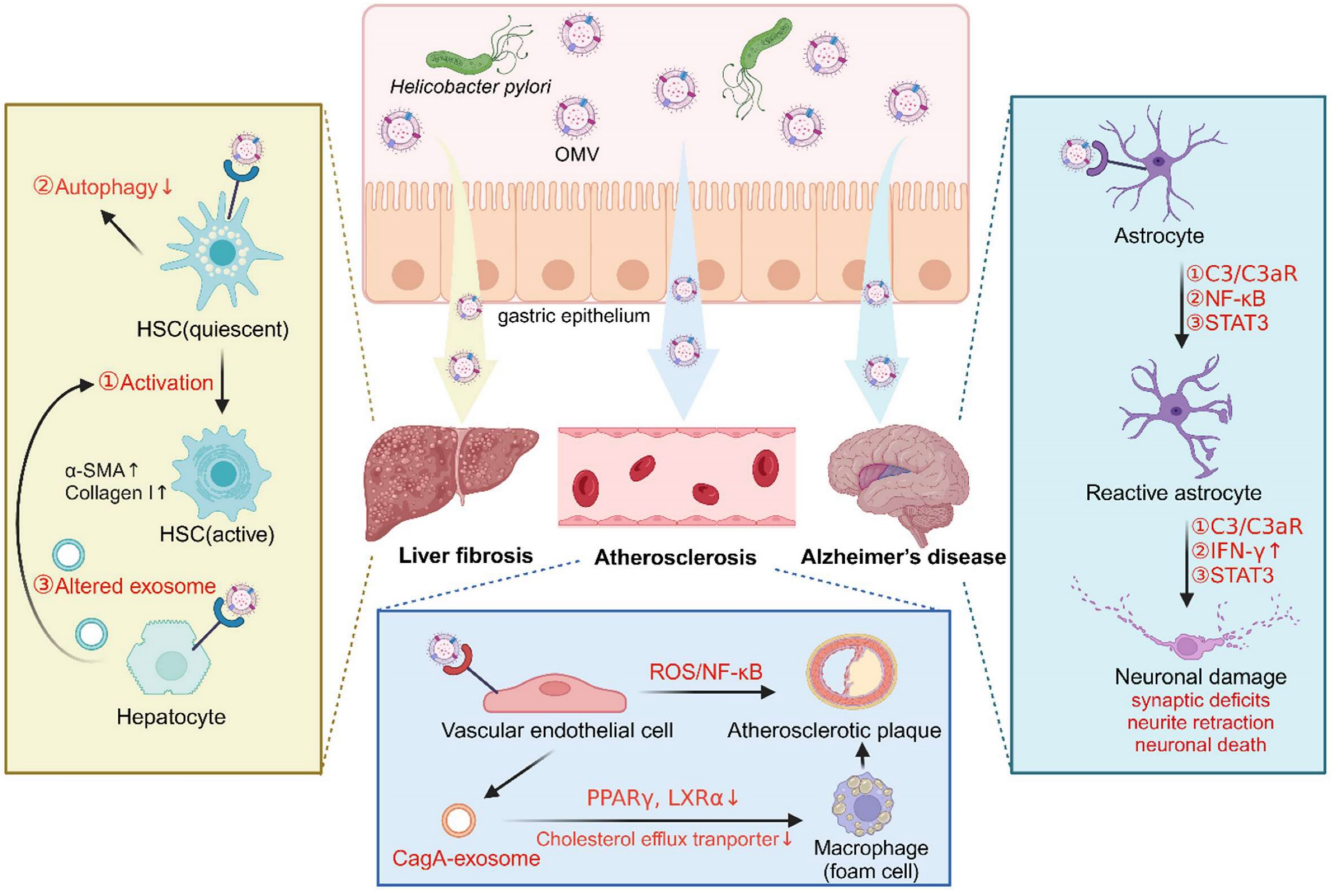
**Role of *H. pylori* OMVs in extra-gastric pathogenesis related to *H. pylori* infection** (created with BioRender.com).** A.** *Helicobacter pylori* OMVs induce the activation of hepatic stellate cells (HSCs) and autophagy inhibition, or indirectly induce altered hepatocyte exosomes and then trigger HSC activation, promoting liver fibrosis. **B.** *Helicobacter pylori* OMVs induce astrocyte reactivity and then cause neuronal damage by activating C3/C3aR, NF-κB or STAT3 signaling pathways, thereby promoting Alzheimer's disease. **C.** *Helicobacter pylori* OMVs either directly impair endothelial function by activating the ROS/ NF-κB signaling, or indirectly stimulate endothelial cells to release CagA-containing exosomes to promote foam cell formation, ultimately accelerating atherosclerotic plaque formation.

In liver fibrosis, an *in vitro* study demonstrated that OMVs isolated from clinical strains of *H. pylori* can trigger the activation of hepatic stellate cells (HSCs) and enhance the expression of hepatic fibrosis markers (α-SMA, E-cadherin, vimentin, snail, and β-catenin) 106. Additionally, exposure of HSCs to *H. pylori* OMVs was found to upregulate autophagy inhibitory makers (PI3K, AKT, and MTOR) while downregulating autophagy core proteins (BECN1, ATG16 and LC3B), suggesting that *H. pylori* OMV-induced autophagy inhibition in HSCs may contribute to the development of *H. pylori*-mediated liver fibrosis 109. Furthermore, another study revealed that hepatocytes treated with *H. pylori* OMVs induced changes to the hepatocyte-derived exosomes, which promoted HSC activation and the progression of liver fibrosis 110 (**Figure 3**).

In Alzheimer's disease, an *in vivo* study demonstrated that OMVs isolated from *H. pylori* strain 26695 can cross the blood-brain barrier to reach the brain, where they were taken up by astrocytes and induced astrocyte reactivation followed by microglial activation via activation of the C3a-C3aR signaling pathway. Reactive phagocytic microglia then actively participated in the elimination of synapses and the phagocytosis of neurons, leading to synaptic deficits and excessive neuronal loss, exacerbating amyloid-β pathology and ultimately, resulting in cognitive decline 107. Interestingly, another study indicated that *H. pylori* OMVs induced astrocyte reactivation, characterized by the production of the neurotoxic factor IFN-γ through activation of the NF-κB signaling pathway, thereby causing neuronal dysfunction including neurite retraction and increased neuronal death 111. Furthermore, an *in vitro* study demonstrated that the STAT3 signaling pathway was involved in the detrimental effects of* H. pylori* OMVs on the brain, thus contributing to the development of Alzheimer's disease 112 (**Figure 3**).

In atherosclerosis, a study reported that OMVs from *H. pylori* strain 26695 accelerated atherosclerotic plaque formation in ApoE ^-/-^ mice, and triggered apoptotic cell death of human umbilical vein endothelial cells (HUVECs) via activation of the ROS/NF-κB signaling pathway 108. Notably, LPS and CagA were identified as two important contributors in these processes, as demonstrated by significantly ameliorated endothelial cell dysfunction upon treatment with LPS-depleting or CagA-negative *H. pylori* OMVs 108. Interestingly, another study revealed that OMVs from *H. pylori* stain PMSS1 stimulated gastric epithelial cells to release CagA-containing exosomes into blood circulation, promoting macrophage-derived foam cell formation and augmenting atherosclerotic plaque growth and instability. Exosomal CagA was reported to downregulate the expression of transcription factors PPARγ and LXRα, leading to the dysfunction of the cholesterol efflux transporter. Consequently, the excessive accumulation of cholesterol in the macrophages induced foam cell formation, thus contributing to the progression of atherosclerosis 113 (**Figure 3**).

Collectively, *H. pylori*-derived OMVs represent nanocarriers of *H. pylori* virulence factors to both local or distant organs, playing a crucial role in the pathogenesis of *H. pylori* infection-related gastric and extra-gastric diseases.

### Exploiting *H. pylori* OMVs as vaccines

#### Potential of *H. pylori* OMVs as vaccine candidates

OMVs share great similarity with their parental bacteria in immunomodulatory components such as lipids, lipoproteins, enzymes, peptidoglycan, and genetic materials (DNA, RNA), by which they can elicit strong humoral and cellular immune responses against pathogens 14, 114. This highly immunogenic potential of bacteria-derived OMVs presents them as promising vaccine candidates against pathogen infection. Currently available OMV vaccines including VA-MENGOC-BC^®^ (1987), MenBVac^®^ (1991), and MeNZB™ (2004) were successfully developed to control meningococcal serogroup B outbreaks in Cuba, Norway and New Zealand, respectively 115-117. Notably, the same OMV used to formulate MeNZB™ was also added to the recently developed MenB vaccine (Bexsero^®^) as an adjuvant in combination with three recombinant proteins (NHBA, NadA, fHBP) to help protect against meningococcal serogroup B infection 118, 119.

Despite more than 30 years of effort in the development of *H. pylori* vaccine, no effective vaccine against *H. pylori* has been developed yet. Lessons learned from the past failures will help in the identification of vaccine candidates with new approaches. The use of *H. pylori* OMVs as potential vaccine candidates may prove to be a promising strategy, as some preliminary cellular and animal studies have provided a basis for their potential protective effects. For instance, an *in vitro* study reported that OMVs from *H. pylori* strain SS1 were able to induce a T helper 2 (Th2) immune response, as demonstrated by high levels of IL-10 and IL-4 in macrophage RAW 264.7 cells. Importantly, compared with the total antigens isolated from *H. pylori* SS1, OMVs showed comparable immunogenic activity and better biocompatibility, hemocompatibility, and safety due to their lack of toxicity 120. An *in vivo* study by Das *et al.* demonstrated that immunization of mice with OMVs from *H. pylori* strain A61C(1) elicited higher level of adaptive immune responses compared to the non-immunized group, which significantly lowered bacterial loads upon challenge with *H. pylori* strain SS1 post immunization 121. In terms of immune responses, another study reported that IFN-γ and IL-17, representative cytokines for Th1 and Th17-mediated immune responses, respectively, were both significantly induced in the spleens of mice immunized with OMVs form *H. pylori* strain HP99 17. Regarding immunization routes, it was observed that intraperitoneal immunization of *H. pylori* OMVs stimulated even higher levels of immunoglobulin (IgG) compared to oral immunization 17. Additionally, Liu *et al.* reported that oral immunization of mice with OMVs from *H. pylori* strain 7.13 induced stronger humoral and mucosal immune responses without inducing mucosal inflammation, compared with *H. pylori* whole cell immunization. This study revealed that *H. pylori* OMVs predominantly induced a Th2-biased immune response, dramatically lowering bacterial loads in a mouse model challenged with *H. pylori* strain SS1 122. Interestingly, when using OMVs from *H. pylori* strain 7.13 as adjuvants during the immunization of mice with 7.13 outer membrane proteins (OMPs) or whole cell vaccine (WCV), more humoral and mucosal immunity was elicited and gastric colonization of *H. pylori* in mice were significantly reduced when compared to the administrations of OMPs or WCV, alone or coupled with standard adjuvant cholera toxin (CT) 123.

In addition to its potential role as a vaccine, *H. pylori* OMVs may also help combat *H. pylori* infection by preventing bacterial adherence to host cells. OMVs mimic their parental bacteria and encapsulate various adhesins, enabling them to inhibit the adhesion of parental bacteria to host cells by competitively binding to the same target sites 124. For instance, *H. pylori* OMV-coated nanoparticles have been reported to adhere to and block *H. pylori* adhesion to AGS cells in a dose-dependent manner. Furthermore, the adherence of these OMV-coated nanoparticles facilitated the detachment of adherent *H. pylori* from mouse stomach tissues 125. The anti-bacterial adhesion efficacy of OMVs can be further enhanced through genetic engineering of bacteria to modulate adhesin expression on the OMVs and by selecting optimal nanoparticle cores with the desired characteristics 126.

#### Challenges in developing *H. pylori* OMVs as vaccines

Current vaccine development using *H. pylori* OMVs as targets faces many challenges including the optimization of OMVs isolation conditions including growth conditions and the selection of a well-characterized strain with an intricate balance between OMV content immunogenicity and toxicity 127. For instance, bacterial LPS is a major content of OMVs in most gram-negative bacteria. It is a very potent activator of immune cells such as monocytes and macrophages, triggering NF-κB and IRF3-mediated pro-inflammatory responses via recognition of the lipid A moiety of LPS by the TLR4/MD2 receptor. This activation is important in initiating and directing the adaptive immune response, which is a highly important vaccine property for effective clearance of bacterial infections 128. However, overly strong reactogenicity triggered by LPS may lead to septic shock. Thus, OMVs are usually prepared with detergent extraction to reduce endotoxin levels, but with some limitations including potential toxicity from residual detergent particles and the unintentional removal of essential LPS-bound OMPs required for immunogenic activity 129. Hence, the use of mutants with defects in lipid A biosynthesis seems to provide a balanced response, generating sufficient adjuvant activity while preventing unwanted effects limiting the vaccine efficacy 24. For instance, *N. meningitidis* Δ*lpxL* mutant was reported to produce LPS with penta- instead of hexa-acylated lipid A, retaining adjuvant activity but with reduced toxicity 130. Unlike other gram-negative bacteria expressing bi-phosphorylated and hexa-acylated LPS,* H. pylori* constitutively modifies its LPS into an under-acylated and dephosphorylated lipid A, effectively reducing its reactogenicity 131, 132. *In vivo* studies using mice and rabbits have shown that *H. pylori* LPS exhibits 500 to 1000 times lower levels of cytotoxicity, mitogenicity, and pyrogenicity as compared to that of other gram-negative pathogens, such as *E. coli* and *S. typhimurium* 133. The constitutively modified LPS structure in *H. pylori* may alleviate the need for detergent extraction or genetic modification of lipid A. However, genetic engineering of *H. pylori* to remove LPS Lewis antigen is required in the development of *H. pylori* OMV vaccines. This is due to the fact that the O-antigen of *H. pylori* LPS usually contains Lewis antigens that resemble human Lewis molecules and blood group antigens, resulting in the potential development of autoimmunity if present 51, 134, 135.

In addition to LPS, other well-known virulence factors present in *H. pylori* OMVs including CagA oncoprotein, VacA vacuolating cytotoxin, and HtrA serine protease (also a known gastric cancer risk factor) also pose a big safety concern for vaccine development 71, 100, 136. One possible option would be to create a genetically-modified strain lacking CagA, VacA, and HtrA virulence factors. However, a drawback of this option may be the reduction or loss of immunogenicity attributed to these virulence factors, resulting in suboptimal immune response. This warrant further investigations. An alternative approach is to select strains containing non-toxigenic virulence factor genotypes or to genetically modify the corresponding genes to generate detoxified or non-virulent subtypes while retaining their immunogenicity. For instance, strains with the VacA subtype s1/m1 exhibit high cytotoxic activity, while those with the subtype s2/m2 show no cytotoxicity 137. This variability necessitates careful selection or genetic modification of non-toxic VacA variants, such as s2/m2, for safe vaccine formulations. Similar approaches can be applied to other virulence factors including CagA and HtrA.

## Summary

In light of the data above, *H. pylori* OMVs play an important role in bacterial colonization, survival and pathogenesis. Due to their nanosized structure, *H. pylori* OMVs can be internalized and translocated across the gastric mucosal epithelial layer to reach the underlying blood circulation system and then migrate to distant cells and organs, causing a plethora of cellular responses and that may explain the association of *H. pylori* infection with extra-gastric diseases. Moreover, the immunogenic, non-replicable, and anti-bacterial adhesion properties of* H. pylori* OMVs make them a desirable vaccine candidate against infection. However, there are many challenges in the development of *H. pylori* OMVs-based vaccines including safety concerns and how to achieve an intricate balance between OMV content immunogenicity and reactogenicity/toxicity. *Helicobacter pylori* OMVs are a double-edged sword. While they may contribute to disease pathogenesis by causing inflammatory tissue damage, they may also hold therapeutic potential in the form of vaccine to help protect against *H. pylori*-related diseases. Future experimental studies are needed for a comprehensive analysis of *H. pylori* OMVs biogenesis and biological functions.

## Abbreviations

MALT: mucosa-associated lymphoid tissue
WHO: world Health Organization
OMVs: outer membrane vesicles
OM: outer membrane
Lpp: lipoprotein
OmpA: outer membrane protein A
LPS: lipopolysaccharide
PQS: pseudomonas quinolone signal
PL: phospholipids
IM: inner membrane
PE: phosphatidylethanolamine
CGs: cholesteryl glucosides
PG: phosphatidylglycerol
PC: phosphatidylcholine
Le^a^: Lewis a
MS: mass spectrometry
T4SS: type IV secretion system
eDNA: extracellular DNA
sncRNA: small non-coding RNA
bOMVs: biofilm OMVs
pOMVs: planktonic OMVs
α-CA: α-class carbonic anhydrase
PBMCs: peripheral blood mononuclear cells
DCs: dendritic cells
HO-1: heme oxygenase-1
HSCs: hepatic stellate cells
Th2: T helper 2
OMPs: outer membrane proteins
WCV: whole cell vaccine
